# Models of care for noncommunicable diseases in primary care: key elements and design in low- and middle-income countries – a scoping review

**DOI:** 10.1080/16549716.2025.2543604

**Published:** 2025-08-28

**Authors:** Mai Eltigany, Laura Drown, Oyetayo Akala, Silvia Ussai, Gene Bukhman, Alarcos Cieza, Alma J. Adler

**Affiliations:** a Noncommunicable Diseases, Rehabilitation and Disability Department, WHO HQ, Geneva, Switzerland; b Center for Integration Science in Global Health Equity, Division of Global Health Equity, Department of Medicine, Brigham and Women’s Hospital, Boston, MA, USA; c Program in Global Noncommunicable Disease and Social Change, Department of Global Health and Social Medicine, Harvard Medical School, Boston, MA, USA

**Keywords:** Noncommunicable diseases, models of care, model elements, theoretical basis, LMICs

## Abstract

Noncommunicable diseases (NCDs) pose a global health challenge, with primary health care’s (PHC) potential to address them underutilized. While efforts to design and implement effective NCDs care models in low- and middle-income countries (LMICs) are increasing, conclusive recommendations are lacking. This scoping review maps literature on NCDs care models in LMICs, examining elements, theoretical foundations, and outcomes to inform the development of context-specific models. Following Arksey and O’Malley’s framework, we searched PubMed, Embase, and Web of Science for articles on NCD care models in LMIC primary care. Two authors independently screened and extracted data using a 40-element framework. Identified models were categorized as implemented or theoretically proposed, analyzed by subcategory, and elements identified and compared. The search and snowballing yielded 1,011 articles. 54 met inclusion criteria, covering 44 models (25 implemented, 19 proposed). Implemented models’ design was largely informed by service delivery gaps analyses. The most frequently included elements in them were education and training of the health workforce, combined self-management and patient education, guidelines/protocol-based approach, information systems, screening, team-based care, combined nurse-led and task shifting/sharing, essential medicines, essential diagnostics, community-level care, standardized referral pathways, and mentoring and supervision. Comparison of frequently included elements in implemented and proposed models showed strong alignment between them and with the recommended interventions in WHO-NCDs technical packages for primary care. This underscores these elements’ relevance in addressing NCD service delivery challenges in LMICs and guiding the reorientation of PHC models to better integrate NCD services. However, further evidence is needed to support implementation.

## Paper Context

- **Main findings**: Service delivery gaps largely informed the design of the implemented noncommunicable diseases (NCDs) care models in low-middle income countries (LMICs). The most frequently included elements in these models were education and training of the health workforce, combined self-management and patient education, guidelines/protocol-based approach, information systems, screening, team-based care, combined nurse-led and task shifting/ sharing, essential medicines, essential diagnostics, community-level care, standardized referral pathways, and mentoring and supervision. A strong alignment was observed between the elements of implemented models and those of theoretically proposed models, as well as with the recommended interventions in the WHO NCD technical packages for primary care.
- **Added knowledge**: The review provided insights into the theoretical basis guiding the design of NCDs care models in LMICs, identified frequently included elements in both implemented models proposed models, and highlighted strong agreement between them, underscoring their relevance in addressing the unique NCDs service delivery challenges in primary care in LMICs.
- **Global health impact for policy and action**: The high level of agreement on key elements in NCDs care models in LMICs suggests they could inform the reorientation of PHC models, enabling more effective integration of NCDs services and addressing persistent service gaps. Nevertheless, further research is needed to validate the elements effectiveness and to inform implementation strategies across diverse settings.

## Background

Noncommunicable diseases (NCDs) are a leading cause of morbidity and mortality worldwide, accounting for approximately 41 million deaths annually, with 17 million of these deaths occurring prematurely (before the age of 70). Four major groups of diseases – cardiovascular diseases, cancers, chronic respiratory diseases, and diabetes – are responsible for 80% of these deaths, underscoring the urgent need for effective management and prevention strategies. The impact of NCDs is particularly severe in resource-limited settings, where 86% of premature deaths occur [1].

The management of NCDs encompasses the full spectrum from screening/early detection to palliative care. Evidence suggests that cost-effective and high-impact essential NCDs interventions can be delivered through a primary health care (PHC) approach [2], which is a whole-of-society approach for maximizing health and well-being through three key components: (a) integrated primary care and essential public health functions, (b) multisectoral policy and action, and (c) empowered people and communities [3].

Despite this evidence, the potential of primary care to deliver comprehensive care for NCDs has not been realized. Indeed, the 2023 Universal Health Coverage (UHC) Monitoring Report indicated slow progress in NCDs services coverage before 2015 and little to no improvement thereafter [4]. This underscores the urgent need to accelerate the integration of NCDs into primary care as a critical step toward achieving UHC.

The inadequate attention to NCDs in low- and middle- income countries (LMICs) stems from chronic underfunding of healthcare and a historical focus on communicable diseases and maternal and child health conditions. Therefore, primary care providers are often untrained and unequipped to promote health by preventing risk factors or screening asymptomatic patients [5]. Additionally, primary care in LMICs often focuses on episodic care designed for acute symptom relief rather than long-term well-being or chronic condition management [6].

To effectively manage NCDs in LMICs, primary care must shift to a comprehensive, integrated approach covering prevention, diagnosis, treatment, rehabilitation, and palliation across the life course [2,5]. PHC-oriented models of care should promote continuous, comprehensive, coordinated and integrated person- and people-centered care, rather than focus on specific diseases (particularly considering the growing recognition of the importance of addressing multimorbidity) [3].

Models of care outline ‘what’ services are provided, ‘for whom’ (target population), ‘by whom’ (workforce), ‘where’ (settings) and ‘how’. The ‘how’ includes strategies, processes, and tools to ensure equity, accessibility, quality, responsiveness, and improved health outcomes. There is no single correct model, instead, models must be tailored to specific contexts. As a result, multiple models can coexist within the same health system. These variations are shaped by needs and feasibility, such as the differences between fragile and stable settings. To remain responsive, models must evolve through continuous monitoring [7]. Although context-specific, successful models can offer valuable insights for planning in similar settings.

Over the past two decades, efforts have been made to improve the organization of primary care services for NCDs and ultimately models of care. Initially, Wagner’s 1999 Chronic Care Model (CCM) was considered effective for guiding the design of services for chronic conditions, however evidence is limited in LMICs as most evidence on CCM is from high income countries [8]. To make CCM more suitable for LMICs, WHO adapted it to include policy environment considerations and the role of the community (WHO Innovative Care for Chronic Conditions (ICCC) Framework) but this framework has rarely been implemented in practice [9].

Subsequently, WHO has introduced technical packages to assist countries in the management of NCDs at the primary care level. These include the 2010 WHO Package of Essential Noncommunicable Disease Interventions for primary care (WHO PEN) and the 2018 WHO HEARTS technical package for cardiovascular disease management in primary care [2,10]. They present a prioritized set of cost-effective interventions for NCDs, designed to maintain acceptable quality of care even in resource-limited settings, and offer guidance on implementation. While these packages do not constitute models of care, they recommend key interventions that can inform models of care development.

This scoping review maps the literature on NCDs models of care in primary care settings in LMICs, identifying the most frequently included elements used to address the unique challenges of NCDs service delivery at the primary care level based on countries’ income levels. Additionally, it describes the theoretical foundations, aims, and outcomes of model implementations, providing deeper insights into the development and evaluation processes of those models. These findings can be used to inform the creation of locally adapted guidance for care models in various LMIC contexts.

## Methods

We conducted a scoping review following Arksey and O’Malley’s methodological framework for scoping studies [11] to map existing evidence on models of care for NCDs in LMICs. The framework involves five key stages: (1) identifying the research question and defining the scope, including inclusion and exclusion criteria; (2) identifying relevant studies; (3) selecting studies based on predefined criteria; (4) charting data using a structured extraction tool; and (5) collating, summarizing, and reporting the findings.

We included models that had either been implemented or were theoretically proposed (not yet implemented). Proposed models were regarded as expert opinions on models of care and served as comparators to assess alignment with implemented models. Each type was analyzed separately to identify areas of convergence in design and elements.

This distinction was based on the understanding that implemented models are typically developed in response to specific health system challenges within defined settings. These models often undergo adaptation during implementation, becoming more comprehensive over time; identifying their core elements can therefore provide valuable guidance for designing models of care in similar contexts. In contrast, proposed models tend to be broader in scope, often designed for LMICs in general, and are predominantly theoretical, with limited empirical validation. For this reason, they were included solely to provide a comparative framework.

### Criteria for considering studies for this review

We utilized the following inclusion criteria: (i) studies conducted in an LMIC as defined by the World Bank at the time of this review [12]; (ii) interventions provided in a primary care setting (community or primary care center, or district hospital, if the district hospital is considered part of primary care based on country-specific definitions); (iii) studies including at least one major NCD: diabetes, hypertension, cardiovascular diseases, cancers, and chronic respiratory diseases (asthma and chronic obstructive pulmonary disease); and (iv) studies that specifically described models of care, defined as how services should be delivered or multi-component intervention [7,13] we did not exclude on language, but we did not include any non-English terms in our search. Systematic reviews were included if they gave recommendations for a proposed model.

We utilized the following exclusion criteria: (i) studies conducted in high-income countries only; (ii) studies conducted only in secondary and/or tertiary health care settings; (iii) studies conducted only in pediatric health care settings (but if a study included both adult and pediatric populations it was included); (iv) studies that did not address any of the four major NCDs (cardiovascular diseases, diabetes, chronic respiratory diseases, and cancer); (v) studies where the primary focus was on a single-component intervention, such as training alone rather than on a model of multi-component interventions; and (vi) study protocols, editorials, or commentaries; studies in emergency settings (humanitarian crises and disasters).

### Search methods for identification of studies

We searched Embase, PubMed, and Web of Science databases using search terms including terms associated with models of care; low- and middle- income countries; noncommunicable diseases including diabetes, cardiovascular disease, hypertension, chronic respiratory disease, and cancer; and primary care (A full search strategy is found in Annex A). The search terms were decided upon collectively by the authors and generated from keywords associated with the research objective.

We searched PubMed, Embase, and Web of Science between 27 September 2023, and 5 October 2023. We included studies published between the period of 1 January 2000, and 3 October 2023. This timeframe was selected in alignment with the emergence of discussions around chronic care models starting with Wagner’s CCM [14].

To complement traditional search methods and conduct a comprehensive review of the literature, authors expanded the pool of relevant literature using both forward and backward snowballing while examining references from systematic reviews.

### Data extraction and synthesis

Search results were imported into the data management software Covidence [15], which removed duplicate articles and allowed the authors (ME, OA, SU) to simultaneously screen titles and abstracts, resolve discrepancies, conduct full article reviews, and generate a PRISMA diagram. Each article was reviewed by two of the three authors mentioned above. Discrepancies were resolved through conversation between the three authors.

The analysis of the models of care (implemented and proposed) was guided by an adapted version of the Foundations Framework for Developing and Reporting New Models of Care for Multimorbidity [16]. The original framework includes 28 elements, grouped into three categories: clinical focus (7), organization of care delivery (13), and support for model delivery (8). The adapted version of the framework had 40 elements and eight sub-elements (Annex B), grouped under the same three aforementioned groups. The additional elements were included to capture CCM elements, elements identified in similar reviews on NCDs models of care in LMICs during the preliminary search (which used the same search terms but was limited to systematic and scoping reviews) and elements noted by the research team during the testing phase of the analytical framework. For the analysis, we developed a data extraction sheet that captured information on the framework foundation (underpinning theory and target population), the adapted version of the models of care elements across three thematic areas, and additional information on the study and the model.

Two authors (ME, OA) conducted data extraction independently, and the findings were consolidated into a single analysis sheet. All discrepancies were resolved through discussion and consensus. Information collected on each model included: title, study type, description of underpinning theory, aim, target population, elements, implementation status, and outcomes, encompassing both clinical (disease metrics) and other outcomes such as system-level outcomes. Additionally, we included information on the country income level, scale of model implementation, number and type of health facilities involved, population covered, and intervention duration. We also collected information on the title, author, year, study design, and the health system context when mentioned.

We analyzed implemented and proposed models separately. Each type of model was further categorized into and analyzed by subgroups according to context to facilitate comparison of elements across settings:

- Implemented models: Countries were categorized by income level into three groups − 1) low-income countries (LICs), 2) lower middle-income countries (L-MICs), and 3) upper middle -income countries (UMICs).
- Proposed models: Models were categorized into two groups − 1) LMICs and sub-Saharan African (SSA) countries, and 2) country-specific recommendations.

### Assessment of risk of bias

This study investigates written descriptions of models of care; hence, we did not conduct a risk of bias assessment. To eliminate bias in the selection process, two out of three researchers (ME, OA, SU) independently screened each article using Covidence [15].

## Results

**Figure 1.**
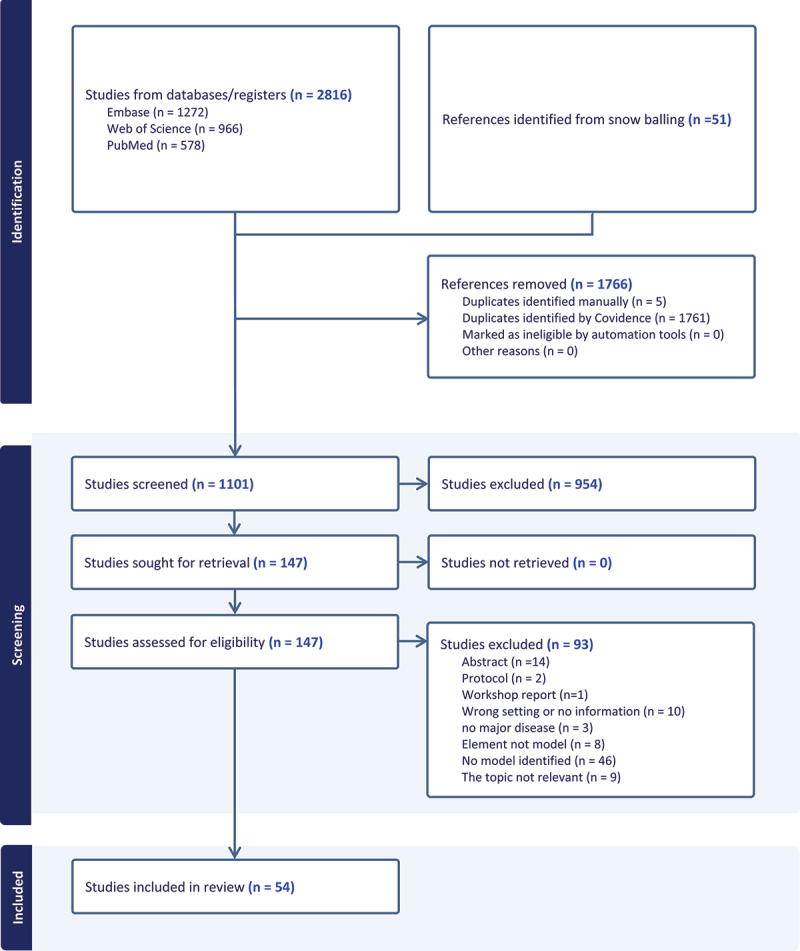
Prisma flow diagram.

Our search yielded 1,272 articles from Embase, 578 articles from PubMed, 966 from the Web of Science databases, and 51 article through snowballing. Upon removing duplicates, there were 1101 unique articles. After title and abstract screening, 147 articles remained. After screening full-texts, 54 articles were found relevant to the study. The final number of articles included in the analysis was 44 unique models from 54 studies, representing 25 implemented models and 19 proposed models (Figure 1). The distribution of articles over time was as follows: eight articles were published before 2010, seven between 2010 and 2014, 22 between 2015 and 2019, and 17 from 2020 onward.

### Implemented models

Over half (14, 56%) of the 25 implemented models were from Africa [17–35]. Three (12%) [36–40] were from South America and eight (32%) [41–51] were from Asia. Most (15, 60%), of the models were implemented in L-MICs. Eight (32%) were from UMICs, and two (8%) in LICs (Table 1). Kenya reported the highest number of implemented models (5), followed by India (3). Mexico, South Africa, and Nigeria each had two implemented models, and the remaining countries (Brazil, China, Cameroon, Colombia, Malaysia, Ethiopia, Ghana, Kenya, Malaysia, Philippines, Uganda, Thailand, and Zimbabwe) each had one.

**Table 1.** Themes and elements of implemented models of care (MOC) by country income and proposed models by region or country.

Themes	Element		Implemented Models								Proposed Models					
			All implemented * (*N*=25)		UMICs (*N*=8)		L-MICs (*N*=15)		LICs (*N*=2)		All proposed (*N*=19)		LMICs/SSA-MOC (*N*=10)		Countries MOC (*N*=9)	
			n	%	n	%	n	%	n	%	n	%	n	%	n	%
Clinical Focus	1	Combined (self-management and patient education)	21	84	7	78	12	86	2	100	13	68	8	80	5	56
	1.1	Self-management support	8	32	5	56	3	21	0	0	9	47	7	70	2	22
	1.2	Patient education	16	64	3	33	11	79	2	100	9	47	5	50	4	44
	2	Adherence support	9	36	4	44	5	36	0	0	7	37	4	40	3	33
	3	Bio-psychosocial (holistic) approach	0	0	0	0	0	0	0	0	0	0	0	0	0	0
	4	Prevention	8	32	3	33	4	29	1	50	9	47	4	40	5	56
	5	Polypharmacy	0	0	0	0	0	0	0	0	0	0	0	0	0	0
	6	Shared decision making	0	0	0	0	0	0	0	0	0	0	0	0	0	0
	7	Mental health and wellbeing	1	4	1	11	0	0	0	0	0	0	0	0	0	0
	8	Guideline/protocol-based approach	20	80	7	78	12	86	1	50	12	63	7	70	5	56
	9	Individualized management plans	3	12	2	22	1	7	0	0	1	5	1	10	0	0
	10	Quality of life	0	0	0	0	0	0	0	0	0	0	0	0	0	0
Organization of care delivery	11	Screening (case finding)	16	64	5	56	10	71	1	50	10	53	5	50	5	56
	11.1	Screening- Facility based	5	20	2	22	3	21	0	0	2	11	2	20	0	0
	11.2	Screening- Community based	7	28	2	22	5	36	0	0	6	32	2	20	4	44
	11.3	Screening-Both	4	16	1	11	2	14	1	50	2	11	1	10	1	11
	12	Case management	0	0	0	0	0	0	0	0	0	0	0	0	0	0
	13	Essential medicines	14	56	5	56	7	50	2	100	15	79	8	80	7	78
	14	Essential diagnostics	13	52	3	33	9	64	1	50	13	68	7	70	6	67
	15	Integration of services	2	8	1	11	1	7	0	0	8	42	4	40	4	44
	16	Defining roles and responsibilities	10	40	3	33	7	50	0	0	2	11	2	20	0	0
	17	Team-based care	15	60	6	67	9	64	0	0	6	32	4	40	2	22
	18	Co-location of services	0	0	0	0	0	0	0	0	1	5	1	10	0	0
	19	Home care	6	24	3	33	3	21	0	0	3	16	2	20	1	11
	20	Decentralized care	5	20	0	0	4	29	1	50	7	37	5	50	2	22
	21	Community level care	13	52	5	56	6	43	2	100	10	53	6	60	4	44
	22	Standardized referral pathway	13	52	4	44	7	50	2	100	8	42	4	40	4	44
	23	Scheduled chronic appointments	12	48	5	56	7	50	0	0	5	26	4	40	1	11
	24	Extended appointment times	0	0	0	0	0	0	0	0	4	21	4	40	0	0
	25	Appointment reminders	4	16	1	11	3	21	0	0	3	16	3	30	0	0
	26	Single professional responsible	0	0	0	0	0	0	0	0	0	0	0	0	0	0
	27	Dedicated NCDs clinic/staff	2	8	0	0	2	14	0	0	2	11	0	0	2	22
	28	New role	3	12	3	33	0	0	0	0	1	5	1	10	0	0
	29	Combined (nurse led and task shifting/sharing)	16	64	4	44	10	71	2	100	10	53	6	60	4	44
	29.1	Nurse-led care	10	40	3	33	5	36	2	100	2	11	1	10	1	11
	29.2	Task shifting/sharing	9	36	2	22	7	50	0	0	10	5	6	60	4	44
	30	Group visits	1	4	1	11	0	0	0	0	1	5	1	10	0	0
	31	24/7 support available	0	0	0	0	0	0	0	0	0	0	0	0	0	0
	32	Education/training of health workforce	22	88	8	89	12	86	2	100	14	74	6	60	8	89
	33	Telehealth	9	36	3	33	6	43	0	0	8	42	4	40	4	44
	34	Funding and payment changes	11	44	5	56	6	43	0	0	8	42	4	40	4	44
	35	Risk stratification tool	11	44	4	44	7	50	0	0	1	5	1	10	0	0
	36	Primary care providers network	1	4	0	0	1	7	0	0	1	5	1	10	0	0
	37	Quality improvement	4	16	3	33	1	7	0	0	5	26	4	40	1	11
	38	Information system-	17	68	5	56	11	79	1	50	14	74	6	60	8	89
	38.1	Information system- general description	5	20	2	22	3	21	0	0	13	68	8	80	5	56
	38.2	Electronic medical record	4	16	0	0	4	29	0	0	7	37	3	30	4	44
	38.3	Paper based medical record	5	20	1	11	3	21	1	50	1	5	1	10	0	0
	38.4	Hybrid medical record	4	16	2	22	2	14	0	0	0	0	0	0	0	0
	39	Health system (mentoring and supervision)	13	52	5	56	6	43	2	100	4	21	1	10	3	33
	40	Space and infrastructure	2	8	0	0	2	14	0	0	1	5	1	10	0	0

Note: LICs, low-income countries; L-MICs lower middle-income countries; LMICs, low- and middle-income countries: UMICs, upper middle -income countries; SSA, Sub-Saharan African.

### Proposed models

The search identified 19 proposed models that have not yet been implemented. Ten of these were recommended for LMICs or SSA [5,6,8,9,52–57], while nine were recommended for specific countries [58–66]. Three of the country-specific models were from India, and Uganda, Tanzania, Botswana, South Africa, Samoa, and China each had one model.

### Summary of framework elements included in models of care

Of the 40 main elements included in the analysis framework, 31 (78%) and 32 (80%) appeared in at least one implemented or proposed model, respectively.

In the implemented models, only 13 elements of the framework were included in LICs, while L-MICs and UMICs models featured 28 and 27 elements, respectively. All elements used in LICs were also included in models in L-MICs. Only one element from LICs was not featured in UMICs, specifically related to health systems – decentralized care. Among the 28 elements implemented in L-MICs or UMICs, there was agreement on 21 elements (75%) (Table 1).

### Models’ elements by theme

#### Clinical focus element

##### Implemented models

The most frequently included elements were combined patient education and self-management support (21, 84%) and the guideline/protocol-based approach (20, 80%), followed by adherence support (9, 36%) and prevention (8, 32%). None of the models included the bio-psychosocial (holistic) approach, polypharmacy, shared decision making, or quality of life elements (highlighted in grey in Table 1).

There were some variations in the elements included across countries of different income levels. The main element of combined patient education and self-management support was common across all income levels (Table 1). However, the sub-element self-management support was more commonly included in UMICs (5, 56%) compared to L-MICs (3, 21%) and LICs (0, 0%) while patient education was more common in L-MICs (11, 79%) and LICs (2, 100%) in contrast to UMICs (3, 33%). Mental health and well-being elements were only included once, in an UMIC model (Table 1). Additionally, it is noteworthy that all models incorporating adherence included self-management support or patient education; some opted to mention both (Table 1).

##### Proposed models

The most frequently included elements in the proposed models under this theme followed a similar order to the implemented models and were as follows: combined patient education and self-management support (13, 68%), a guideline or protocol-based approach (12, 63%), prevention (9, 47%), adherence support (7, 37%), and individualized management plans (1, 5%). Five elements were not reflected in the models: shared decision-making, polypharmacy, a bio-psycho-social (holistic) approach, quality of life, and mental health and wellbeing (Table 1).

#### Organization of care elements

##### Implemented models

The most commonly included elements were screening, and combined nurse-led task and shifting/sharing (both 16, 64%), followed by team-based care (15, 60%). Integration of services and dedicated NCDs clinic/staff (both 2, 8%), new role (3, 12%), and group visits (1, 4%) were included the least frequently. The elements not included were case management, co-location of services, extended appointment times, single professional responsible, and 24/7 support available (Table 1).

Regarding the variation in model elements by income level, combined nurse-led care and task shifting/sharing and decentralized health systems were mostly included in models implemented in L-MICs. Specifically, combined nurse-led care and task shifting/sharing was present in 10 models (71%) in L-MICs and both models (100%) in LICs, but only in four models (44%) in UMICs. Decentralized care was included in four models (29%) in L-MICs and one model (50%) in LICs but was absent in UMICs. Conversely, new roles and group visits were exclusively incorporated in models implemented in UMICs (Table 1).

There was also variation in the screening approach: seven models (28%) opted for community-based screening, five applied facility-based screening (20%), and four combined both approaches (16%) (Table 1).

##### Proposed models

The proposed models prioritized similar elements to the implemented models. Essential medicines was the most frequently included element reported in 15 (79%) of the proposed models, followed by essential diagnostic (13, 68%), combined nurse-led and task shifting/sharing, screening, and community-level care (10, 53%) each. Community screening was the most common form (6, 32%) (Table 1).

Less frequently included elements in proposed models included defining roles and responsibilities and dedicated NCDs clinics/staff, which were in two models (11%) each. Co-location of services, group visits, and new roles were included in one (5%) model each. Elements not reflected include having a single professional responsible, case management, and 24/7 support availability (Table 1).

#### Support for model delivery element

##### Implemented models

Most models included education/training of the health workforce (22, 88%), followed by information systems (17, 68%), which could be broadly mentioned or specified as electronic records, paper-based, or hybrid. Other popular elements included health system (mentoring and supervision) (13, 52%), risk stratification tools (11, 44%), and funding and payment changes (11, 44%) (Table 1).

The listed elements supporting model delivery were predominantly included in models for UMICs and L-MICs. Models in LICs included only three elements: education/training of the health workforce, information system, and health systems (mentoring and supervision).

##### Proposed models

The two most included elements in the implemented models were prioritized education and training of the health workforce and combined systems (each 14, 74%). These were followed by telehealth and funding/payment changes (8, 42%), quality improvement (5, 26%), mentoring and supervision (4, 21%), and risk stratification tools, primary care provider networks, and space and infrastructure (1, 5%) (Table 1).

### Comparison of the most frequently included elements in implemented and proposed models

To identify the most frequently included elements, we examined elements that were included in at least 50% of the models. This analysis revealed that 12 elements were frequently included in the implemented models, while nine were frequently included in the proposed models.

Comparison between both the implemented and proposed models revealed complete overlap: all nine frequently included elements in the proposed models were also among the 12 most commonly included in the implemented models. The three additional elements prioritized only in the implemented models were team-based care, standardized referral pathways, and mentoring and supervision (Table 2).

**Table 2.** Comparison of the most frequently included elements in implemented and proposed models.

Implemented models (N=25)				Proposed models (N=19)			
	Element	n	%		Element	n	%
1	Education/training of health workforce	22	88	1	Essential medicines	15	79
2	Combined self-management and patient education	21	84	2	Education/training of health workforce	14	74
3	Guideline/protocol-based approach	20	80	3	information system	14	74
4	information system	17	68	4	Combined self-management support and patient education	13	68
5	Screening (case finding)	16	64	5	Essential diagnostics	13	68
6	Combined nurse led and task shifting/sharing	16	64	6	Guideline/protocol-based approach	12	63
7	Team based care	15	60	7	Screening (case finding)	10	53
8	Essential medicines	14	56	8	Community level care	10	53
9	Essential diagnostics	13	52	9	Combined nurse led and task shifting/sharing	10	53
10	Community level care	13	52				
11	Standardized referral pathway	13	52				
12	Mentoring and supervision	13	52				

### Models’ target population by disease

The diseases included in the implemented models were hypertension, diabetes, dyslipidemia/hyperlipidemia, epilepsy, cardiovascular diseases, renal disease, stroke, obesity, and asthma. Over half of the implemented models (14, 56%) included more than one disease.

Similarly, most proposed models (15, 79%) targeted multiple conditions. Other conditions addressed included chronic obstructive pulmonary disease (COPD).

### Theoretical basis: aims, values, and assumptions

#### Models’ aims

The purposes of the implemented models were generally well-defined, with most sharing a common goal of improving the effectiveness and quality of NCDs care [17,25,28–31,34,37,38,43,46,48]. While some models prioritized cost reduction through increased service efficiency, lower expenses, or cost-effective approaches [20,32,33,37,41], others emphasized improving access to care [19,21,26,27].

In contrast, the proposed models had broader objectives, aiming to develop contextually relevant models for efficient and effective service delivery at scale, address care gaps, scale up the response to the NCDs epidemic, and reorient the system towards chronic care [6,55,56,60,62,63,65].

#### Theoretical basis and assumption

The design of implemented models was primarily guided by identifying gaps in NCDs services and barriers to care within the country’s context and developing interventions to address them. Some HIV care models were influenced by and adapted from specific care models, such as HIV care, the CCM, the ICCC, and the recommended interventions in WHO PEN. Others used a more general approach, such as a health system enhancement strategy or a PHC model emphasizing a family- and community-oriented approach. Additionally, some models focused on innovations such as mHealth and technology integration in the PHC model or learning from previous experiences in the country or similar contexts. In a few cases, the theoretical basis for the model design was not identified (Table 3).

**Table 3.** Types of NCDs included in the implemented models and theoretical basis.

	Implemented models								Proposed					
Single condition or integrated	LICs (*N*=2)		L-MICs (*N*=15)		UMICs (*N*=8)		Total (*N*=25)		LMICs/SSA (*N*=10)		Countries (*N*=9)		Total (*N*=19)	
	*n*	*%*	*n*	*%*	*n*	*%*	*n*	*%*	*n*	*%*	*n*	*%*	*n*	*%*
Single	0	0	7	47	4	50	11	44	2	20	2	22	4	21
Integrated	2	100	8	53	4	50	14	56	8	80	7	78	15	79
**Type of NCDs included by condition in all models**														
Hypertension	1	50	12	80	5	63	18	72	1	10	0	0	1	5
Diabetes	2	100	11	73	6	75	19	76	3	30	2	22	5	26
Dyslipidaemia/Hyperlipidaemia	0	0	0	0	2	25	2	8	0	0	0	0	0	0
Obesity	0	0	1	7	1	13	2	8	0	0	0	0	0	0
CVD	0	0	2	13	0	0	2	8	1	10	0	0	1	5
Epilepsy	1	50	1	7	0	0	2	8	0	0	0	0	0	0
Renal disease/proteinuria	0	0	1	7	1	13	2	8	0	0	0	0	0	0
Asthma/COPD	0	0	1	7	0	0	1	4	1	10	0	0	1	5
CVD/stroke	0	0	0	0	1	13	1	4	0	0	0	0	0	0
**Assumptions for model development**														
Analysis of barriers and gaps in NCDs services	0	0	4	27	2	25	6	24	0	0	3	33	3	16
HIV care models/DSD*^+^	0	0	3	20	0	0	3	12	4	40	1	11	5	26
CCM	0	0	1	7	2	25	3	12	1	10	1	11	2	11
ICCC & WHO PEN*	0	0	1	7	0	0	1	4	0	0	0	0	0	0
ICCC*^+^	0	0	0	0	0	0	0	0	0	0	2	22	2	11
TB DOTS	0	0	0	0	0	0	0	0	1	10	0	0	1	5
TB DOTS and CCM^+^	0	0	0	0	0	0	0	0	1	10	0	0	1	5
Health system/PHC-oriented approach/PHC redesign*^+^	0	0	0	0	2	25	2	8	1	10	2	22	3	16
Previous experience in the country/region	1	50	0	0	1	13	2	8	0	0	0	0	0	0
Innovations like mHealth and technology in the PHC model	0	0	3	20	1	13	4	16	0	0	0	0	0	0
Not identified	1	50	2	13	1	13	4	16	2	20	0	0	2	11

CCM, Chronic Care Model: COPD chronic obstructive pulmonary disease; CVD, Cardiovascular disease; DSD, Differentiated Service Delivery; HIV Human Immunodeficiency Virus; ICCC Innovative Care for Chronic Conditions framework; health, Mobile health; WHO PEN, Package of Essential Noncommunicable (NCDs) Disease Interventions: PHC Primary Health Care; TB-DOTS, Tuberculosis treatment strategy ((Directly Observed Treatment, Short-course).

*Barriers analyses conducted to further inform the model development.

+Systematic and literature reviews conducted to further inform the model development.

Similarly, the proposed models that provided recommendations for SSA or LMICs utilized various approaches (Table 3). Of these 10 models, four (40%) articles used HIV care models as examples for NCD care. Two articles (20%) were based on the CCM, with one incorporating elements from both the CCM and the TB-DOTS (directly observed treatment, short-course) model. One article (10%) was informed by TB-DOTS alone, another article (10%) examined redesigning primary care, drawing on emerging literature to address evolving NCDs challenges (Table 3).

In addition to the theoretical basis, several articles conducted a gap/barriers analysis for NCDs services in primary care before formulating assumptions for their models while others relied on finding from systematic or literature reviews. In two articles (20%), the assumptions for the model were not identified (Table 3).

Like the implemented models, three country-specific proposed models referred to an analysis of barriers in their assumptions (3, 33%), followed by the ICCC (2, 22%), and primary care or health system approach (2, 22%) then an HIV care model, and CCM (each at 1, 11%). The development process was further supplemented by barriers analysis and literature review in several models (Table 3).

### Evaluation

Out of the 25 implemented models, 24 (96%) reported some aspect of effectiveness of the model, typically aimed at measuring changes brought about by introducing the model of care. Five models (20%) reported both clinical and non-clinical outcomes, nine (36%) focused on clinical outcomes only, and 10 (40%) reported non-clinical outcomes only.

All 14 models that evaluated clinical outcomes reported improvements, with significant improvements in 11 (79%). Of the remaining papers, two (14%) reported non-significant positive outcomes and one (7%) reported significant positive outcomes in both the intervention and control groups. The most frequently used metrics were blood pressure control and diabetes control, measured by HbA1c or glucose levels. Other indicators included low-density lipoprotein (LDL), high-density lipoprotein (HDL), cholesterol, dyslipidemia, days of hospital admissions, asthma attacks, cardiovascular risk scores, fasting blood glucose, body mass index (BMI), waist-to-hip ratio, and stroke-associated morbidity and mortality (Table 4).

**Table 4.** Models of care (MOC) evaluation.

				Clinical outcome	Non-clinical outcomes
MOC No.	Author	Country	Study type	Summary effect	Summary effect
1	Otieno HA	Ghana & Kenya	Cohort	Positive (significant)*	N/A
2	Husin M	Malaysia	Quasi-experimental controlled study	Positive**	Overall model effect-Mixed Positive (significant) for this study
	Low LL		Mixed-methods observational	N/A	N/A
	Johari MZ (2020)		Qualitative	N/A	Positive
	Wong WJ		Quasi-experimental	N/A	Mixed***
	Ministry of Health, Malaysia		Government report	NA	N/A
3	Katende D	Uganda	Cross-sectional	N/A	Positive (significant)
4	Tapia-Conyer R	Mexico	Mixed-methods descriptive	N/A	N/A
	Gallardo-Rincón H		Cohort	Positive (significant)	
5	Duan K	Mexico	Quasi-experimental	Positive (significant)	N/A
6	Mamo Y	Ethiopia	Descriptive	N/A	Positive
7	Frieden M	Zimbabwe	Descriptive	N/A	Positive
8	Ku GMV	Philippines	Cohort	Positive (significant)	Positive (significant)
9	Nelissen HE	Nigeria	Mixed methods	Positive (significant)	Positive (reported by the two studies)
	Cremers AL		Mixed methods	N/A	
10	Hendriks ME	Nigeria	Cohort	positive (Significant)	Positive
11	Ng’endo K	Kenya	Cohort	N/A	Positive
12	Ogola EN	Kenya	Cluster-randomized controlled trial	N/A	Positive
13	Shannon GD	Kenya	Mixed methods	N/A	Mixed
14	Cabral NL	Brazil	Cohort	Positive (significant)	N/A
15	Chao J	China	Randomized controlled trial	Positive	Positive
16	Lall D	India	Case experimental teven	N/A	Feasibility/Partially feasible
17	Gill GV	South Africa	Cohort	Positive (significant)	N/A
18	Prabhakaran D	India	Randomized controlled trial	Positive (significant)	N/A
19	Jindal D	India	Descriptive	N/A	N/A
20	Schwalm J-D	Colombia Malaysia	Cluster-randomized controlled trial	Positive (significant)	N/A
21	Kengne AP (2009A)	Cameroon	Descriptive	N/A	N/A
	Kengne AP (2009B)		Cohort	Positive (significant)	
	Kengne AP (2008)	Cameroon	Cohort		
	Kengne AP (2009C)	Cameroon	Cohort		
22	Van de Vijver S (2013)	Kenya	Descriptive	N/A	N/A
	Van de Vijver S (2016)		Quasi-experimental	Positive (significant both arms)	
23	Vedanthan R	Kenya	Descriptive	N/A	Positive
24	Katz I	South Africa	Cohort	N/A	Mixed
25	Chaowalaksakun P	Thailand	Qualitative	N/A	Positive

*Positive (significant): A positive impact that is statistically significant.

**Positive: A positive effect that was either not statistically significant or reflected only as a percentage change without a statistical significance test applied.

***Mixed: The assessment showed both positive and negative effects.

Non-clinical outcomes were assessed through both quantitative and qualitative measures. Of the 15 models, two (13%) showed significant positive changes: one in NCDs service readiness and the other in the Patient Assessment of Chronic Illness Care (PACIC). Nine (60%) models demonstrated improvements in access, quality of care, retention, people-centeredness, satisfaction, and enrolment while one (7%) demonstrated that the model was practical, well-suited to the context; however, the interventions were only partially implemented at all sites except one. (Table 4). Three (20%) models had mixed results: the first showed significant improvement in care process but discrepancies in job satisfaction, with pharmacists and nutritionists more satisfied than stressed nurses. The second model improved medicine availability but revealed affordability issues related to expenses of the treatment monitoring, medicines, and travel for diabetic patients due to low implementation rates of the access improvement aspects of the intervention. The third model effectively helped the primary care nurses detect advanced disease and ensured early referrals, enhancing their knowledge. However, weaknesses included inadequate follow-up of patients and a workforce that was overworked, poorly supported, and faced high staff turnover (Table 4). For more information on the measured variables, study settings, and participants, see Annex D.

### Sustainability

Sustainability was inconsistently addressed across the implemented models of care. Only two studies explicitly evaluated the sustainability of interventions [18,26], while a third described the model as both appropriate and sustainable [27], and a fourth inferred sustainability through the demonstrated impact of a nationwide cardiovascular disease prevention program [39]. Some models integrated sustainability considerations during the design phase [24,32,34,46], whereas others identified ongoing challenges – such as inadequate health system inputs, administrative inefficiencies, and reliance on external funding which could challenge scale up efforts despite positive outcomes [19,21,23,35,37,40,41]. In several cases, no information related to sustainability was reported.

Among the two studies that formally assessed sustainability, one from a low-income country reported that hypertension and diabetes services remained largely functional four years after external funding had ended, indicating the potential for sustained service delivery beyond donor support [18]. In contrast, the second study, conducted in a lower middle-income country found that while insulin access had improved, limited advancements were made in areas such as early detection and quality of care. This imbalance presented ongoing challenges to both sustainability and scalability of the intervention [26].

## Discussion

In this scoping review, we identified and analyzed the elements of 25 implemented models of care in LMICs and compared them to 19 proposed models based on expert and national recommendations for LMICs. Most implemented models originated from L-MICs, with fewer from UMICs and only two from LICs. Publications were primarily from recent years, indicating an increasing emphasis on enhancing service delivery for NCDs, given their growing burden. Most models were evaluated quantitatively or qualitatively.

The design of the implemented models of care was primarily guided by service delivery gaps analysis, whereas the proposed models were mostly informed by learning from chronic disease models such as HIV care models and the CCM with some considering contextual barriers to NCDs care. A few also reflected on the ICCC, the TB-DOTS model and the PHC approach. These findings align with a systematic review in SSA which identified models and frameworks of primary care interventions for priority NCDs in LMICs to guide development of an a priori framework for data synthesis [52]. The review identified two models – the TB DOTS model of care approach for tuberculosis and the adapted CCM – for L-MICs as theoretical bases. It was noted that the TB DOTS model, previously applied to scaling up HIV services, is now adapted for the management of NCDs in Sub-Saharan Africa [52].

This review analyzed implemented and proposed models of care separately before comparing the most frequently included elements. This approach was based on the understanding that implemented models, shaped by real-world application, tend to be more context-specific and comprehensive. In contrast, proposed models offer valuable theoretical insights but lack empirical validation. Their inclusion therefore served primarily to provide a comparative framework.

The comparison revealed notable similarities in the elements commonly included in both types of models. All nine elements frequently included in the proposed models were also frequently included elements in implemented models, which included an additional three elements. This consensus suggests widespread agreement on the elements to consider when developing models of care to address the challenges of NCDs service delivery at the primary care level in LMICs or to reorient models of care to effectively integrate NCDs in these settings.

Models in L-MICs and UMICs included twice as many elements compared to the models from LICs. Constraints in LICs and the limited number of models from this context may have contributed to this disparity. The inclusion of all elements from models in LICs in those in L-MICs suggests common challenges and potential for cross-learning. Moreover, the considerable similarity between the elements implemented in L-MICs and UMICs underscores their relevance in both settings, thereby facilitating knowledge exchange.

The prioritized elements closely align with the TB DOTS model, incorporating four of its components: case detection, standardized treatment, a regular and uninterrupted drug supply, and a standardized recording and reporting system, but not the fifth, political commitment [67]. This alignment underscores the importance of drawing lessons from other successful chronic disease models and programs like those addressing TB and HIV [6, 20, 34, 64), which have been noted for their effectively decentralized services in low-resource settings. It also stresses the importance of engaging local and national governments, along with health authorities, to leverage existing evidence in developing context-specific and locally adapted models of care.

The comparison of frequently included elements in implemented models with recommended interventions in the two WHO technical packages for noncommunicable diseases in primary care (the WHO PEN and HEARTS Technical Package for Cardiovascular Diseases Management) shows a considerable level of alignment. The WHO PEN provides guideline/protocol-based approaches to managing NCDs to facilitate disease management and the training of care providers. It includes recommendations on self-care, early detection, information systems, essential medicines and diagnostics, mentoring and supervision and the development of standardized referral pathways, which are among the most frequently included elements in implemented models. However, the WHO PEN does not provide recommendations on combined nurse-led and task-shifting strategies, community-level care, and team-based care, which are often included in models for L-MICs and LICs.

While the WHO PEN recommends early detection and opportunistic screening of cardiovascular disease risk factors by focusing on eligible patients, such as measuring blood pressure for all adults aged 18 and above at seeking care at health facilities, many models have opted for community-based screening to identify and engage cases. Some supporters of community-based screening highlight its success in the rollout of HIV care [20], while others argue that it is not cost-effective and favor facility-based screening [32]. Therefore, further evidence is essential to guide screening practices, particularly for common NCDs like diabetes and hypertension, which can be asymptomatic in the early stages of the disease.

The HEARTS Technical Package for Cardiovascular Diseases Management in Primary Care builds on WHO PEN, and both emphasize self-care, with strategies aligned with patient beliefs to enhance adherence. In line with WHO recommendations, several models in UMICs tend to prioritize self-management support, while models in L-MICs and LICs often prioritize health education. This difference could be related to human resource constraints.

The HEARTS Technical Package recommends team-based care, which is frequently included in L-MIC and UMIC models but often absent in LIC models, likely due to constraints in LIC settings [10]. An article discussing a nurse-led model for managing hypertension and diabetes in rural LMICs reflects on this point, arguing that the Western approach, which relies on multidisciplinary teams and specialized, resource-intensive care, may not be suitable for resource-limited settings where nurses serve as the primary healthcare providers [20].

In this article, we combined task-shifting or sharing and nurse-led care, as they all point to non-physician staff taking a role in managing of NCDs despite the variation in the definition of the terms. This element was more commonly included in L-MICs and LICs, likely due to human resource constraints. This finding aligns with a scoping review on models for integrating NCDs at the primary care level, which indicated that many models included decentralization of services and task shifting to lower cadre providers [68].

The majority of the models were evaluated quantitatively or qualitatively, with significant variation in the strength of evidence. Most models showed a positive impact on the set targets. However, three models reported mixed results, with two of these attributed to staff dissatisfaction among nurses, who felt undervalued, unsupported, unauthorized to perform tasks, and overworked. This contrasts with a model that reported a positive impact on nurses, where interventions included addressing nurse shortages through adequate staffing and strengthening the administrative role to effectively implement changes, among other measures.

The variability in the strength of evidence underscores the importance of more systematically embedding implementation research within models of care implementation, complemented by rigorous evaluation methodologies. Such an approach can more effectively guide the contextual adaptation of models in LMICs and generate more data on their effectiveness. In addition, evaluating the sustainability of interventions and proactively addressing related challenges are essential for informing their scalability.

Some elements were less frequently included in the implemented and proposed models, such as funding and payment changes, telehealth, and quality of care. Despite not ranking among the most included elements, these interventions remain important for sustainable change and delivering high-quality services and could be highly relevant in some contexts.

Some elements, such as case management, quality of life, and polypharmacy, were not included in any models (implemented or proposed). The search strategy could have influenced this, as it focused on models that included at least one major NCDs but didn’t include terms such as multimorbidity, for which a case manager might be required, or the public health approach for services delivery in LMICs, which favors standardized protocol.

### Study limitations

Our study includes few models of care from LICs, which restricts the generalizability of our findings to these settings. The unique challenges and resource constraints in LICs may necessitate different approaches to care that are not fully captured in our analysis. This study was limited to a specific set of diseases and to studies that specified a model of care, so some studies that did not specify these diseases or models of care may have been missed. Additionally, as this review was restricted to published studies, we may have missed unpublished examples. While we attempted to overcome this by hand searching references, it is likely that we have missed some studies.

While several models in this study have been thoroughly evaluated, some have not undergone assessment of clinical outcomes and one did not conduct any form of evaluation. This incomplete evaluation limits our ability to fully understand their effectiveness and scalability. Consequently, the conclusions drawn about these models should be interpreted with caution.

The proposed models of care we included were derived from articles that either intended to propose new approaches for reorienting care models to better deliver NCDs services at the primary care level, or offered recommendations based on assessments of existing services or reflections on gaps in NCDs care. This selective inclusion may overlook other relevant models and innovative approaches proposed in different contexts. Therefore, the list presented in our study is not exhaustive but reflects the models identified through our search strategy.

## Conclusion

This review identified the most frequently included elements in implemented NCDs models of care that have been applied in LMICs and could inform practice in these contexts. Furthermore, comparing these elements with those commonly featured in the theoretically proposed models revealed strong alignment, both between the two model types and with the recommended interventions in the WHO NCD technical packages for primary care. Given the limited evidence on the most effective elements of NCDs models of care in LMICs, this alignment underscores key elements importance in addressing the unique challenges of delivering NCDs services in these settings and suggests they could inform the reorientation of PHC models of care to better integrate NCDs services.

Despite this consensus, further research is needed to determine which elements are most effective in practice, as the evidence base for implemented models remains limited. Context-specific factors must also be carefully considered to ensure that models of care are adaptable and sustainable across diverse health systems. Additionally, future efforts should prioritize gathering evidence on the implementation strategies for elements such as screening, self-management support, task-shifting/sharing, nurse-led care, and team-based care, to inform the practice.

## Annexes Annex A. PubMed search strategy

The following search string/MESH terms were used:“model* of care”[All Fields] OR “service delivery”[All Fields] OR “service organization”[All Fields] OR “healthcare approach*”[All Fields] AND “low and middle income countr*” OR “low to middle income countr*” OR “low income countr*” OR “middle income countr*” OR “low resource” OR “developing countr*” OR “Africa” OR “Asia” OR “Latin America” OR “afghanistan”[MeSH Terms] OR “afghanistan”[All Fields] OR “afghanistan s”[All Fields] OR (“angola”[MeSH Terms] OR “angola”[All Fields] OR “angola s”[All Fields]) OR (“albania”[MeSH Terms] OR “albania”[All Fields]) OR (“argentina”[MeSH Terms] OR “argentina”[All Fields] OR “argentina s”[All Fields] OR “argentinae”[All Fields]) OR (“armenia”[MeSH Terms] OR “armenia”[All Fields]) OR (“american samoa”[MeSH Terms] OR (“american”[All Fields] AND “samoa”[All Fields]) OR “american samoa”[All Fields]) OR (“azerbaijan”[MeSH Terms] OR “azerbaijan”[All Fields]) OR (“burundi”[MeSH Terms] OR “burundi”[All Fields]) OR (“benin”[MeSH Terms] OR “benin”[All Fields] OR “benin s”[All Fields]) OR (“burkina faso”[MeSH Terms] OR (“burkina”[All Fields] AND “faso”[All Fields]) OR “burkina faso”[All Fields]) OR (“bangladesh”[MeSH Terms] OR “bangladesh”[All Fields] OR “bangladesh s”[All Fields]) OR (“bulgaria”[MeSH Terms] OR “bulgaria”[All Fields]) OR (“bosnia and herzegovina”[MeSH Terms] OR (“bosnia”[All Fields] AND “herzegovina”[All Fields]) OR “bosnia and herzegovina”[All Fields] OR “bosnia”[All Fields]) OR (“republic of belarus”[MeSH Terms] OR (“republic”[All Fields] AND “belarus”[All Fields]) OR “republic of belarus”[All Fields] OR “belarus”[All Fields]) OR (“belize”[MeSH Terms] OR “belize”[All Fields]) OR (“bolivia”[MeSH Terms] OR “bolivia”[All Fields]) OR (“brazil”[MeSH Terms] OR “brazil”[All Fields] OR “brazil s”[All Fields] OR “brazils”[All Fields]) OR (“bhutan”[MeSH Terms] OR “bhutan”[All Fields] OR “bhutan s”[All Fields]) OR (“botswana”[MeSH Terms] OR “botswana”[All Fields] OR “botswana s”[All Fields]) OR (“central african republic”[MeSH Terms] OR (“central”[All Fields] AND “african”[All Fields] AND “republic”[All Fields]) OR “central african republic”[All Fields]) OR (“china”[MeSH Terms] OR “china”[All Fields] OR “china s”[All Fields] OR “chinas”[All Fields]) OR (“cote d ivoire”[MeSH Terms] OR (“cote”[All Fields] AND “d ivoire”[All Fields]) OR “cote d ivoire”[All Fields]) OR (“cameroon”[MeSH Terms] OR “cameroon”[All Fields] OR “cameroons”[All Fields] OR “cameroon s”[All Fields]) OR (“congo”[MeSH Terms] OR “congo”[All Fields]) OR “Democratic Republic of the Congo”[All Fields] OR (“colombia”[MeSH Terms] OR “colombia”[All Fields] OR “colombia s”[All Fields]) OR (“comoros”[MeSH Terms] OR “comoros”[All Fields] OR “comoro”[All Fields]) OR (“cabo verde”[MeSH Terms] OR (“cabo”[All Fields] AND “verde”[All Fields]) OR “cabo verde”[All Fields]) OR (“costa rica”[MeSH Terms] OR (“costa”[All Fields] AND “rica”[All Fields]) OR “costa rica”[All Fields]) OR (“cuba”[MeSH Terms] OR “cuba”[All Fields]) OR (“djibouti”[MeSH Terms] OR “djibouti”[All Fields]) OR (“dominica”[MeSH Terms] OR “dominica”[All Fields]) OR (“dominican republic”[MeSH Terms] OR (“dominican”[All Fields] AND “republic”[All Fields]) OR “dominican republic”[All Fields]) OR (“algeria”[MeSH Terms] OR “algeria”[All Fields]) OR (“ecuador”[MeSH Terms] OR “ecuador”[All Fields] OR “ecuador s”[All Fields]) OR (“egypt”[MeSH Terms] OR “egypt”[All Fields] OR “egypt s”[All Fields]) OR (“eritrea”[MeSH Terms] OR “eritrea”[All Fields]) OR (“ethiopia”[MeSH Terms] OR “ethiopia”[All Fields] OR “ethiopia s”[All Fields]) OR (“fiji”[MeSH Terms] OR “fiji”[All Fields]) OR (“micronesia”[MeSH Terms] OR “micronesia”[All Fields]) OR (“gabon”[MeSH Terms] OR “gabon”[All Fields]) OR (“georgia”[MeSH Terms] OR “georgia”[All Fields] OR “georgia republic”[MeSH Terms] OR (“georgia”[All Fields] AND “republic”[All Fields]) OR “georgia republic”[All Fields] OR “georgia s”[All Fields]) OR (“ghana”[MeSH Terms] OR “ghana”[All Fields] OR “ghana s”[All Fields]) OR (“guinea”[MeSH Terms] OR “guinea”[All Fields] OR “guinea s”[All Fields] OR “guineas”[All Fields]) OR (“gambia”[MeSH Terms] OR “gambia”[All Fields] OR “gambia s”[All Fields]) OR (“guinea bissau”[MeSH Terms] OR “guinea bissau”[All Fields] OR (“guinea”[All Fields] AND “bissau”[All Fields]) OR “guinea bissau”[All Fields]) OR (“equatorial guinea”[MeSH Terms] OR (“equatorial”[All Fields] AND “guinea”[All Fields]) OR “equatorial guinea”[All Fields]) OR (“grenada”[MeSH Terms] OR “grenada”[All Fields]) OR (“guatemala”[MeSH Terms] OR “guatemala”[All Fields] OR “guatemala s”[All Fields]) OR (“guyana”[MeSH Terms] OR “guyana”[All Fields]) OR (“honduras”[MeSH Terms] OR “honduras”[All Fields]) OR (“haiti”[MeSH Terms] OR “haiti”[All Fields] OR “haiti s”[All Fields]) OR (“indonesia”[MeSH Terms] OR “indonesia”[All Fields] OR “indonesia s”[All Fields] OR “indonesias”[All Fields]) OR (“india”[MeSH Terms] OR “india”[All Fields] OR “india s”[All Fields] OR “indias”[All Fields]) OR (“iran”[MeSH Terms] OR “iran”[All Fields]) OR (“iraq”[MeSH Terms] OR “iraq”[All Fields]) OR (“jamaica”[MeSH Terms] OR “jamaica”[All Fields] OR “jamaica s”[All Fields]) OR (“jordan”[MeSH Terms] OR “jordan”[All Fields]) OR (“kazakhstan”[MeSH Terms] OR “kazakhstan”[All Fields] OR “kazakhstan s”[All Fields]) OR (“kenya”[MeSH Terms] OR “kenya”[All Fields] OR “kenya s”[All Fields]) OR (“kyrgyzstan”[MeSH Terms] OR “kyrgyzstan”[All Fields] OR (“kyrgyz”[All Fields] AND “republic”[All Fields]) OR “kyrgyz republic”[All Fields]) OR (“cambodia”[MeSH Terms] OR “cambodia”[All Fields] OR “cambodia s”[All Fields]) OR (“micronesia”[MeSH Terms] OR “micronesia”[All Fields] OR “kiribati”[All Fields]) OR (“laos”[MeSH Terms] OR “laos”[All Fields] OR (“lao”[All Fields] AND “people s”[All Fields] AND “democratic”[All Fields] AND “republic”[All Fields]) OR “lao people s democratic republic”[All Fields]) OR (“laos”[MeSH Terms] OR “laos”[All Fields]) OR (“lebanon”[MeSH Terms] OR “lebanon”[All Fields] OR “lebanon s”[All Fields]) OR (“liberia”[MeSH Terms] OR “liberia”[All Fields] OR “liberia s”[All Fields]) OR (“libya”[MeSH Terms] OR “libya”[All Fields]) OR (“saint lucia”[MeSH Terms] OR (“saint”[All Fields] AND “lucia”[All Fields]) OR “saint lucia”[All Fields] OR (“st”[All Fields] AND “lucia”[All Fields]) OR “st lucia”[All Fields]) OR (“sri lanka”[MeSH Terms] OR (“sri”[All Fields] AND “lanka”[All Fields]) OR “sri lanka”[All Fields]) OR (“lesotho”[MeSH Terms] OR “lesotho”[All Fields]) OR (“morocco”[MeSH Terms] OR “morocco”[All Fields]) OR (“moldova”[MeSH Terms] OR “moldova”[All Fields]) OR (“madagascar”[MeSH Terms] OR “madagascar”[All Fields] OR “madagascar s”[All Fields]) OR (“indian ocean islands”[MeSH Terms] OR (“indian”[All Fields] AND “ocean”[All Fields] AND “islands”[All Fields]) OR “indian ocean islands”[All Fields] OR “maldives”[All Fields] OR “maldive”[All Fields]) OR (“mexico”[MeSH Terms] OR “mexico”[All Fields] OR “mexico s”[All Fields] OR “mexicos”[All Fields]) OR (“micronesia”[MeSH Terms] OR “micronesia”[All Fields] OR (“marshall”[All Fields] AND “islands”[All Fields]) OR “marshall islands”[All Fields]) OR (“republic of north macedonia”[MeSH Terms] OR (“republic”[All Fields] AND “north”[All Fields] AND “macedonia”[All Fields]) OR “republic of north macedonia”[All Fields] OR (“north”[All Fields] AND “macedonia”[All Fields]) OR “north macedonia”[All Fields]) OR (“mali”[MeSH Terms] OR “mali”[All Fields]) OR (“myanmar”[MeSH Terms] OR “myanmar”[All Fields] OR “myanmar s”[All Fields] OR “myanmars”[All Fields]) OR (“montenegro”[MeSH Terms] OR “montenegro”[All Fields]) OR (“mongolia”[MeSH Terms] OR “mongolia”[All Fields] OR “mongolia s”[All Fields]) OR (“mozambique”[MeSH Terms] OR “mozambique”[All Fields] OR “mozambique s”[All Fields]) OR (“mauritania”[MeSH Terms] OR “mauritania”[All Fields]) OR (“malawi”[MeSH Terms] OR “malawi”[All Fields] OR “malawi s”[All Fields]) OR (“malaysia”[MeSH Terms] OR “malaysia”[All Fields] OR “malaysia s”[All Fields]) OR (“namibia”[MeSH Terms] OR “namibia”[All Fields]) OR (“niger”[MeSH Terms] OR “niger”[All Fields]) OR (“nigeria”[MeSH Terms] OR “nigeria”[All Fields] OR “nigeria s”[All Fields]) OR (“nicaragua”[MeSH Terms] OR “nicaragua”[All Fields] OR “nicaragua s”[All Fields]) OR (“nepal”[MeSH Terms] OR “nepal”[All Fields] OR “nepal s”[All Fields]) OR (“pakistan”[MeSH Terms] OR “pakistan”[All Fields] OR “pakistan s”[All Fields]) OR (“peru”[MeSH Terms] OR “peru”[All Fields]) OR (“philippine”[All Fields] OR “philippines”[MeSH Terms] OR “philippines”[All Fields]) OR (“papua new guinea”[MeSH Terms] OR (“papua”[All Fields] AND “new”[All Fields] AND “guinea”[All Fields]) OR “papua new guinea”[All Fields]) OR (“democratic people s republic of korea”[MeSH Terms] OR (“democratic”[All Fields] AND “people s”[All Fields] AND “republic”[All Fields] AND “korea”[All Fields]) OR “democratic people s republic of korea”[All Fields]) OR (“paraguai”[All Fields] OR “paraguay”[MeSH Terms] OR “paraguay”[All Fields]) OR “Gaza”[All Fields] OR (“russia”[MeSH Terms] OR “russia”[All Fields] OR (“russian”[All Fields] AND “federation”[All Fields]) OR “russian federation”[All Fields]) OR (“russia”[MeSH Terms] OR “russia”[All Fields] OR “russia s”[All Fields] OR “russias”[All Fields]) OR (“rwanda”[MeSH Terms] OR “rwanda”[All Fields] OR “rwanda s”[All Fields]) OR (“sudan”[MeSH Terms] OR “sudan”[All Fields] OR “sudans”[All Fields] OR “sudan s”[All Fields]) OR (“senegal”[MeSH Terms] OR “senegal”[All Fields] OR “senegal s”[All Fields]) OR (“melanesia”[MeSH Terms] OR “melanesia”[All Fields] OR (“solomon”[All Fields] AND “islands”[All Fields]) OR “solomon islands”[All Fields]) OR (“sierra leone”[MeSH Terms] OR (“sierra”[All Fields] AND “leone”[All Fields]) OR “sierra leone”[All Fields]) OR (“el salvador”[MeSH Terms] OR (“el”[All Fields] AND “salvador”[All Fields]) OR “el salvador”[All Fields]) OR (“somalia”[MeSH Terms] OR “somalia”[All Fields]) OR (“serbia”[MeSH Terms] OR “serbia”[All Fields]) OR (“south sudan”[MeSH Terms] OR (“south”[All Fields] AND “sudan”[All Fields]) OR “south sudan”[All Fields]) OR (“Sao”[All Fields] AND “Tome”[All Fields]) OR (“suriname”[MeSH Terms] OR “suriname”[All Fields] OR “surinam”[All Fields]) OR (“eswatini”[MeSH Terms] OR “eswatini”[All Fields]) OR (“syria”[MeSH Terms] OR “syria”[All Fields] OR (“syrian”[All Fields] AND “arab”[All Fields] AND “republic”[All Fields]) OR “syrian arab republic”[All Fields]) OR (“syria”[MeSH Terms] OR “syria”[All Fields] OR “syria s”[All Fields]) OR (“chad”[MeSH Terms] OR “chad”[All Fields]) OR (“togo”[MeSH Terms] OR “togo”[All Fields]) OR (“thailand”[MeSH Terms] OR “thailand”[All Fields] OR “thailand s”[All Fields]) OR (“tajikistan”[MeSH Terms] OR “tajikistan”[All Fields]) OR (“turkmenistan”[MeSH Terms] OR “turkmenistan”[All Fields]) OR (“timor leste”[MeSH Terms] OR “timor leste”[All Fields] OR (“timor”[All Fields] AND “leste”[All Fields]) OR “timor leste”[All Fields]) OR (“tonga”[MeSH Terms] OR “tonga”[All Fields] OR “tonga s”[All Fields]) OR (“tunisia”[MeSH Terms] OR “tunisia”[All Fields]) OR (“turkey”[MeSH Terms] OR “turkey”[All Fields] OR “turkey s”[All Fields] OR “turkeys”[MeSH Terms] OR “turkeys”[All Fields]) OR (“micronesia”[MeSH Terms] OR “micronesia”[All Fields] OR “tuvalu”[All Fields]) OR (“tanzania”[MeSH Terms] OR “tanzania”[All Fields] OR “tanzania s”[All Fields]) OR (“uganda”[MeSH Terms] OR “uganda”[All Fields] OR “uganda s”[All Fields]) OR (“ukraine”[MeSH Terms] OR “ukraine”[All Fields] OR “ukraine s”[All Fields]) OR (“uzbekistan”[MeSH Terms] OR “uzbekistan”[All Fields]) OR (vincent, st[Investigator] OR st vincent[Author] OR st vincent[Investigator]) OR (“saint vincent and the grenadines”[MeSH Terms] OR (“saint”[All Fields] AND “vincent”[All Fields] AND “grenadines”[All Fields]) OR “saint vincent and the grenadines”[All Fields] OR “grenadines”[All Fields]) OR (“venezuela”[MeSH Terms] OR “venezuela”[All Fields] OR “venezuela s”[All Fields]) OR (“vietnam”[MeSH Terms] OR “vietnam”[All Fields] OR “vietnam s”[All Fields]) OR (“vanuatu”[MeSH Terms] OR “vanuatu”[All Fields]) OR (“samoa”[MeSH Terms] OR “samoa”[All Fields] OR “samoas”[All Fields]) OR (“kosovo”[MeSH Terms] OR “kosovo”[All Fields] OR “kosovo s”[All Fields]) OR (“yemen”[MeSH Terms] OR “yemen”[All Fields]) OR (“south africa”[MeSH Terms] OR (“south”[All Fields] AND “africa”[All Fields]) OR “south africa”[All Fields]) OR (“zambia”[MeSH Terms] OR “zambia”[All Fields] OR “zambia s”[All Fields]) OR (“zimbabwe”[MeSH Terms] OR “zimbabwe”[All Fields] OR “zimbabwe s”[All Fields]) OR ((“poverty”[MeSH Terms] OR “poverty”[All Fields] OR (“low”[All Fields] AND “income”[All Fields]) OR “low income”[All Fields]) AND (“countries”[All Fields] OR “country”[All Fields] OR “country s”[All Fields] OR “countrys”[All Fields])) OR ((“middle”[All Fields] OR “middles”[All Fields]) AND (“income”[MeSH Terms] OR “income”[All Fields] OR “incomes”[All Fields]) AND (“countries”[All Fields] OR “country”[All Fields] OR “country s”[All Fields] OR “countrys”[All Fields])) OR (“developing countries”[MeSH Terms] OR (“developing”[All Fields] AND “countries”[All Fields]) OR “developing countries”[All Fields] OR (“developing”[All Fields] AND “country”[All Fields]) OR “developing country”[All Fields])

And

((((“noncommunicable disease*”[All Fields] OR “non communicable disease*”[All Fields] OR “NCD”[All Fields] OR (“NCDs”[All Fields] AND (“diabete”[All Fields] OR “diabetes mellitus”[MeSH Terms] OR (“diabetes”[All Fields] AND “mellitus”[All Fields]) OR “diabetes mellitus”[All Fields] OR “diabetes”[All Fields] OR “diabetes insipidus”[MeSH Terms] OR (“diabetes”[All Fields] AND “insipidus”[All Fields]) OR “diabetes insipidus”[All Fields] OR “diabetic”[All Fields] OR “diabetics”[All Fields] OR “diabets”[All Fields]))) AND “cardiovascular disease*”[All Fields]) OR (“hypertense”[All Fields] OR “hypertension”[MeSH Terms] OR “hypertension”[All Fields] OR “hypertension s”[All Fields] OR “hypertensions”[All Fields] OR “hypertensive”[All Fields] OR “hypertensive s”[All Fields] OR “hypertensives”[All Fields])) AND “chronic respiratory disease*”[All Fields]) AND (“cancer s”[All Fields] OR “cancerated”[All Fields] OR “canceration”[All Fields] OR “cancerization”[All Fields] OR “cancerized”[All Fields] OR “cancerous”[All Fields] OR “neoplasms”[MeSH Terms] OR “neoplasms”[All Fields] OR “cancer”[All Fields] OR “cancers”[All Fields]) AND (“primary health care”[All Fields] OR “primary care”[All Fields])

## Annex B. The foundations framework elements vs the adapted version of the elements

**Table:**

Foundations Framework (1) elements	Adapted versions of elements	
	Main elements	Sub-elements
**Theme: Clinical focus**		
Self-management support	Combined – self-management support and patient education)	• Self-management support • Patient education
Biopsychosocial approach	Bio-psychosocial (holistic) approach	
Prevention focus	Prevention	
Polypharmacy attention	Polypharmacy (regular use of 5 or more medications at the same time (2))	
Shared decision making	Shared decision making (between the care provide and the patient)	
Mental health focus	Mental health and wellbeing	
Guidelines/protocols focus	Guideline/protocol-based approach	
	Adherence support	
	Individualized management plans	
	Quality of life	
**Theme: Organization of care delivery**		
Case/care management	Case management (defined as a health care process in which a professional helps a patient or client develop a plan that coordinates and integrates the support services that the patient/client needs to optimize the healthcare and psychosocial possible goals and outcomes (3))	
Integration social/community care (community care)	Community level care	
Integration secondary care	Standardized referral pathway	
Multidisciplinary team/collaborative care	Team-based care: defined here as a care team composed of two members or more who work collaboratively to deliver care	
Co-location of services	Co-location of services (in the same facility)	
Home care	Home care	
Scheduled chronic appointments	Scheduled chronic appointments (provide appointments for regular follow up)	
Extended appointments	Extended appointment times (long intervals between the visits)	
Group visits	Group visits (activities conducted in the clinic for a group of patients)	
24/7 support available	24/7 support available	
Trained lay navigator/coach		
Single professional responsible	Single professional responsible	
Nurse led	Combined nurse led and task sharing (task-sharing is defined as is the reassignment of clinical and non-clinical tasks from one level or type of health worker to another so that health services can be provided more efficiently or effectively (4))	• Nurse led care • Task shifting
	Defining roles and responsibilities	
	Dedicated NCDs clinic/staff: Assigning NCD management responsibilities to specific staff, such as a diabetes nurse, or establishing a clinic dedicated exclusively to NCD services.	
	New role: Assigning existing staff a new role that had not previously been implemented in the clinic.	
	Appointment reminders	
	Screening	• Screening at facility • Screening in community • Both
	Health systems (essential medicines)	
	Health systems (essential diagnostics)	
	Integration of services (same settings and providers)	
**Support for model delivery**		
Upskilling of primary care workforce	Education/training of health workforce	
Education of professionals		
Funding/payment change	Funding and payment changes	
Risk stratification tool	Risk stratification tool	
Primary care provider network	Primary care provider network	
Telephone management	Telehealth (including telephone consultations and use of technology in health)	
Telehealth		
Clinical IT linkage		
	Quality improvement	
	Information system	• Information system- general description • Electronic medical record • Paper based medical record • Hybrid medical record
	Health system (mentoring and supervision)	
	Space and infrastructure	

1. Stokes J, Man MS, Guthrie B, Mercer SW, Salisbury C, Bower P. The Foundations Framework for Developing and Reporting New Models of Care for Multimorbidity. Ann Fam Med. 2017;15(6):570–7. doi: 10.1370/afm.2150. PubMed PMID: 29133498; PubMed Central PMCID: PMC5683871.
2. Varghese D, Ishida C, Patel P, Koya HH. Polypharmacy. StatPearls. Treasure Island: StatPearls Publishing; 2024.
3. Giardino AP, De Jesus O. Case Management. StatPearls. Treasure Island (FL)2024.
4. World Health Organization. HEARTS, Technical package for cardiovascular disease management in primary health care, Team Based Care. Geneva: World Health Organization, 2018.

## Annex C. Elements of implemented and proposed models of care

### A) Implemented models elements by model of care

**Table:**

Theme	Author Element	Otieno HA	Wong WJ	Katende D	Tapia-Conyer R	Duan K	Mamo Y	Frieden M		Ku G.M.V	Nelissen, HE	Hendriks, ME	Kuria N	Ogola EN
	Combined - Self-management & patient education	X	X	X	X	X	X	X		X	X		X	X
	Self-management support		X		X			X		X				
	Patient education	X		X		X	X	X			X		X	X
	Decision support (adherence)		X			X					X		X	
	Bio-psychosocial (holistic) approach													
	Prevention		X	X	X						X	X		X
	Polypharmacy													
	Shared decision making													
	Mental health and wellbeing													
	Guideline or protocol-based approach	X	X	X	X			X		X	X	X	X	X
	Individualized management plans	X			X									
	Quality of life													
	Author Element	Otieno HA	Wong WJ	Katende D	Tapia-Conyer R	Duan K	Mamo Y	Frieden M		Ku G.M.V	Nelissen, HE	Hendriks, ME	Kuria N	Ogola EN
Organization of Care Delivery	Screening (case finding)	X	X	X	X	X		X		X	X		X	X
	Case management													
	Health systems (essential medicines)			X	X	X	X	X					X	X
	Health systems (essential diagnostics)	X		X	X			X			X	X		X
	Integration of services		X					X						
	Defining roles and responsibilities	X	X								X		X	
	Team based care	X	X		X	X				X	X		X	
	Co-location of services													
	Home care	X			X	X				X			X	
	Health systems (decentralized care)	X					X	X			X			
	Community level care	X		X	X	X	X			X	X		X	
	Decision support (standardized referral pathway)	X	X	X	X	X	X	X					X	X
	Scheduled chronic appointments	X	X			X		X		X	X	X	X	
	Extended appointment times													
	Decision support (appointment reminders)		X											
	Single professional responsible													
	Dedicated NCD clinic/staff													
	New role		X			X								
	Combined - Nurse led & task shifting		X	X			X	X		X	X			X
	Nurse-led care			X			X	X						X
	Task shifting/sharing		X					X		X	X			
	Group visits													
	24/7 support available													
	Author Element	Otieno HA	Wong WJ	Katende D	Tapia-Conyer R	Duan K	Mamo Y	Frieden M		Ku G.M.V	Nelissen, HE	Hendriks, ME	Kuria N	Ogola EN
Support for Model Delivery	Education/Training of health workforce		X	X	X	X	X	X		X	X	X		X
	Telehealth	X								X	X			
	Funding and payment changes				X	X					X	X	X	
	Risk stratification tool	X	X		X			X		X		X	X	
	Primary care providers network	X												
	Quality improvement		X		X			X						
	Combined (Information system)	X	X	X	X			X		X	X	X	X	
	Information system (general description)									X				
	Electronic medical record	X												
	Paper based medical record			X				X				X		
	Hybrid medical record		X		X						X		X	
	Health system (mentoring and supervision)		X	X		X	X	X		X				
	Space and Infrastructure												X	

Theme	Author Element	Otieno HA	Wong WJ	Katende D	Tapia-Conyer R	Duan K	Mamo Y	Frieden M		Ku G.M.V	Nelissen, HE	Hendriks, ME	Kuria N	Ogola EN
Theme	Author Element	Shannon GD	Cabral NL	Chao J	Lall D	Gill GV	Prabhakaran D	Jindal D	Schwalm JD	Kengne AP	van de Vijver S	Vedanthan R	Katz I	Chaowalaksakun P
	Combined - Self-management & patient education	X		X		X	X	X	X	X	X	X	X	
	Self-management support Patient education	X		X		X							X	
		X				X	X	X	X	X	X	X	X	
	Decision support (adherence)						X		X	X	X			
	Bio-psychosocial (holistic) approach													
	Prevention			X	X									
	Polypharmacy													
	Shared decision making													
	Mental health and wellbeing			X										
	Guideline or protocol-based approach		X		X	X	X	X	X	X	X		X	X
	Individualized management plans			X										
	Quality of life													
	Author Element	Shannon GD	Cabral NL	Chao J	Lall D	Gill GV	Prabhakaran D	Jindal D	Schwalm JD	Kengne AP	van de Vijver S	Vedanthan R	Katz I	Chaowalaksakun P
Organization of Care Delivery	Screening (case finding)	X							X	X	X	X	X	
	Case management													
	Health systems (essential medicines)	X				X			X	X	X	X	X	
	Health systems (essential diagnostics)	X				X			X	X	X	X		
	Integration of services													
	Defining roles and responsibilities				X		X	X	X			X		X
	Team based care	X	X		X		X	X	X			X		X
	Co-location of services													
	Home care								X					
	Health systems (decentralized care)											X		
	Community level care		X		X				X			X		X
	Decision support (standardized referral pathway)							X			X	X	X	
	Scheduled chronic appointments		X	X						X				X
	Extended appointment times													
	Decision support (appointment reminders)				X		X				X			
	Single professional responsible													
	Dedicated NCD clinic/staff						X			X				
	New role													X
	Combined - Nurse led & task shifting				X	X	X	X	X	X	X	X	X	
	Nurse-led care					X			X	X	X	X	X	
	Task shifting/sharing				X	X	X	X				X		
	Group visits			X										
	24/7 support available													
	Author Element	Shannon GD	Cabral NL	Chao J	Lall D	Gill GV	Prabhakaran D	Jindal D	Schwalm JD	Kengne AP	van de Vijver S	Vedanthan R	Katz I	Chaowalaksakun P
Support for Model Delivery	Education/Training of health workforce	X	X	X	X	X	X	X	X	X	X	X		X
	Telehealth		X	X			X	X	X			X		
	Funding and payment changes					X			X	X	X	X	X	
	Risk stratification tool						X		X		X		X	
	Primary care providers network													
	Quality improvement													X
	Combined (Information system)			X	X		X	X		X		X	X	X
	Information system (general description)			X	X							X		X
	Electronic medical record						X	X				X		
	Paper based medical record									X			X	
	Hybrid medical record													
	Health system (mentoring and supervision)	X					X		X	X		X	X	X
	Space and Infrastructure											X		

### B) Proposed model elements by model of care

**Table:**

Clinical focus	Author Element	Kane J	Tisdale RL	Birabwa, C	Pati MK	Lall, D	Van Olmen J	Bygrave H	Mwangome M	Ke X.-T	Jayanna K
	Combined - Self-management & patient education	X			X	X	X			X	X
	Self-management support					X	X			X	
	Patient education	X			X		X			X	X
	adherence	X			X						X
	Bio-psychosocial										
	Prevention	X			X					X	
	Polypharmacy										
	Shared decision making										
	Mental health and wellbeing										
	Guideline or protocol-based approach	X	X	X	X	X	X	X	X		X
	Individualized management plans					X					
	Quality of life										
Organization of care delivery	Author Element	Kane J	Tisdale RL	Birabwa, C	Pati MK	Lall, D	Van Olmen J	Bygrave H	Mwangome M	Ke X.-T	Jayanna K
	Screening (case finding)	X	X		X					X	X
	Case management										
	Health systems (essential medicines)	X	X	X	X	X	X	X	X	X	X
	Health systems (essential diagnostics)	X	X	X	X	X	X	X	X		X
	Integration of services				X	X	X		X		
	Defining roles and responsibilities					X					
	Multidisciplinary approach/Collaborative care				X	X					
	Co-location of services										
	Home care									X	
	Health systems (decentralized care)	X	X			X	X		X		
	Community level care		X		X	X	X				
	Decision support (standardized referral pathway)	X			X	X					X
	Scheduled chronic appointments	X				X	X				X
	Extended appointment times	X	X					X			
	Decision support (appointment reminders)	X				X					
	Single professional responsible										
	Dedicated NCD clinic/staff									X	X
	New role										
	Combined - nurse led and task shifting	X	X				X		X		
	Nurse-led care										
	Task shifting/sharing	X	X				X		X	X	
	Group visits		X								
	24/7 support available										
Support for model delivery	Author Element	Kane J	Tisdale RL	Birabwa, C	Pati MK	Lall, D	Van Olmen J	Bygrave H	Mwangome M	Ke X.-T	Jayanna K
	Education/Training of health workforce		X	X	X	X			X	X	
	Telehealth									X	X
	Funding and payment changes		X		X					X	
	Risk stratification tool										
	Primary care providers network					X					
	Quality improvement	X		X		X					
	Combined (Information system)		X	X	X	X		X	X	X	X
	Information system (general description)		X	X	X	X		X	X	X	X
	Electronic medical record		X					X			X
	Paper based medical record										
	Hybrid medical record										
	Health system (mentoring and supervision)			X						X	
	Space and Infrastructure						X				

Clinical focus	Author Element	Fraser-Hurt N	Reid M	Kruk M	Singh K	Highton P	Maimela E	Rabkin M	Nath A	Harries AD
	Combined - Self-management & patient education	X		X	X	X	X	X		X
	Self-management support			X	X	X	X	X		X
	Patient education	X			X			X		
	adherence	X		X				X		X
	Bio-psychosocial									
	Prevention	X	X	X	X	X	X			
	Polypharmacy									
	Shared decision making									
	Mental health and wellbeing									
	Guideline or protocol-based approach		X		X					X
	Individualized management plans									
	Quality of life									
Organization of care delivery	Author Element	Fraser-Hurt N	Reid M	Kruk M	Singh K	Highton P	Maimela E	Rabkin M	Nath A	Harries AD
	Screening (case finding)	X		X	X		X			X
	Case management									
	Health systems (essential medicines)	X		X	X				X	X
	Health systems (essential diagnostics)			X	X		X		X	
	Integration of services		X	X		X	X			
	Defining roles and responsibilities							X		
	Multidisciplinary approach/Collaborative care		X	X	X			X		
	Co-location of services							X		
	Home care							X		
	Health systems (decentralized care)	X						X		
	Community level care	X		X		X	X	X	X	
	Decision support (standardized referral pathway)	X			X	X	X			
	Scheduled chronic appointments							X		
	Extended appointment times				X					
	Decision support (appointment reminders)			X						
	Single professional responsible									
	Dedicated NCD clinic/staff									
	New role							X		
	Combined - nurse led and task shifting	X		X	X		X	X	X	
	Nurse-led care				X		X			
	Task shifting/sharing	X		X	X			X	X	
	Group visits									
	24/7 support available									
Support for model delivery	Author Element	Fraser-Hurt N	Reid M	Kruk M	Singh K	Highton P	Maimela E	Rabkin M	Nath A	Harries AD
	Education/Training of health workforce	X	X	X	X	X	X	X	X	
	Telehealth	X		X	X	X		X	X	
	Funding and payment changes			X		X	X		X	X
	Risk stratification tool				X					
	Primary care providers network									
	Quality improvement				X			X		
	Combined (Information system)	X	X		X	X	X			X
	Information system (general description)	X	X		X	X	X			
	Electronic medical record	X	X							X
	Paper based medical record									X
	Hybrid medical record									
	Health system (mentoring and supervision)						X			X
	Space and Infrastructure									

## Annex D. Models of Care (MOC) Evaluation

**Table:**

MOC -No.	Author	Country	Study type	Number of facilities	Study participants	Conditions	Clinical outcomes		Non-clinical outcomes	
							Outcomes assessed	Summary effect	Outcomes assessed	Summary effect
1	Otieno HA	Ghana Kenya	Cohort	9	1266	Hypertension (HTN)	Proportion of patients with controlled blood pressure (BP)	Positive (significant)	None	N/A
2	Husin M	Malaysia	Quasi-experimental controlled study	40 (20intervention, 20 control)	12017	Type 2 diabetes (T2DM)	Hemoglobin A1C (HbA1c) Blood Pressure (BP) Low-density lipoprotein (LDL) High-density lipoprotein (HDL)	Positive, non-significan author indicated-longer duration required to achieve impact	Significant relative changes in eight out of 14 process of care measures in the intervention group	Mixed-Overall effect Positive (significant) for this study
	Low LL		Mixed-methods observational	40 (20 intervention, 20 control)	Not reported	T2DM,hypertension (HTN)	None	N/A	None	N/A
	Johari MZ (2020)		Qualitative	20	35	Not specified	None	N/A	Access Quality of care People-centeredness	Positive
	Wong WJ		Quasi-experimental	40 (20 intervention, 20 control)	Baseline: 1042 Post-intervention: 1215	T2DM, HTN	None	N/A	Provider satisfaction (positive and negative- varied by their profession and roles. Nurses reported higher stress levels and being “under-respected)	Mixed
	Ministry of Health, Malaysia		Government report			Above	Above	NA	Above	N/A
3	Katende D	Uganda	Cross-sectional	22	Not reported	T2DM, HTN	None	N/A	Sustainability 4 years following the end of the intervention measured by assessing NCDs service availability and readiness.	Positive (significant)
4	Tapia-Conyer R	Mexico	Mixed-methods descriptive	130 PHCs in 25 out of the 32 Mexican states	37 PHC HCPs shadowed and interviewed; 224 PHC HCP self-administeredquestionnaires; 68 in-depth interviews	NCDs (specifically T2DM, others unspecified)	None	N/A	None	N/A
	Gallardo-Rincón H		Cohort		743000	Diabetes mellitus (DM), HTN, obesity, dyslipidemia	Screening prevalence Diabetic control	Positive (significant)	None	N/A
5	Duan K	Mexico	Quasi-experimental	9	Not reported	DM, HTN	Diabetes control Hypertension control	Positive (significant)	None	N/A
6	Mamo Y	Ethiopia	Descriptive	5	5056	NCDs, particularly DM and epilepsy	None	N/A	Enrollment in care Retention in care	Positive
7	Frieden M	Zimbabwe	Descriptive	11	3094	DM, HTN	None	N/A	Enrollment in care	Positive
8	Ku GMV	Philippines	Cohort	2	203	DM	HbA1c Proportion of patients with optimally controlled diabetes	Positive (significant)	Patient Assessment of Chronic Illness Care (PACIC)	Positive (significant)
9	Nelissen HE	Nigeria	Mixed-methods	5	336	HTN	BP	Positive (significant)	Patient satisfaction Provider satisfaction Retention in care Quality of care Feasibility	Positive
	Cremers AL		Mixed-methods		30 hypertensive patients; 15 providers; structured interviews with 328 patients		None	N/A	Access	Positive
10	Hendriks ME	Nigeria	Cohort	1	349	Cardiovascular disease (CVD), HTN, DM, renal disease	BP Blood glucose primary prevention, smoking and obesity (As part of assessing the quality of care measured using the adjusted cardiovascular care quality indicators of the United Kingdom National Health Services Quality and Outccome Framework (QOF).	Positive (significant)(Significant for BP control- non significant for blood glucose control)	Quality of care: −Process indicators	Positive (:High quality of care score comparable to high-income countries)
11	Ng’endo K	Kenya	Cohort	10	785	HTN	None	N/A	Visit compliance Medication adherence	Positive
12	Ogola EN	Kenya	Cluster-randomized controlled trial	148	Not reported	HTN	None	N/A	Provider knowledge Quality of care Service utilization	Positive (All improved, however, the provider knowledge showed partial improvement)
13	Shannon GD	Kenya	Mixed methods	35	Not reported	DM	None	N/A	Medicine availability Affordability Sustainability Scalability	Mixed (Improved medicine availability but low implementation rates of the access improvement aspects))
14	Cabral NL	Brazil	Cohort	56	241	Cardiovascular diseases (CVD)-stroke, myocardial infarction.	Stroke All-cause death	Positive (significant)	None	N/A
15	Chao J	China	Randomized controlled trial	1	100	T2DM	Body mass index (BMI) Waist-to-hip ratio Diastolic BP Fasting blood glucose Reduction in days of hospital admissions	Positive	Health knowledge Self-evaluated psychological health status Self-evaluated health status Physical activity duration	Positive
16	Lall D	India	Case experimental design	3	Not reported	DM, HTN	None	N/A	Feasibility	Partially feasible
17	Gill GV	South Africa	Cohort	14	284	DM	HbA1c	Positive (significant)	None	N/A
18	Prabhakaran D	India	Randomized controlled trial	40	3698	DM, HTN	Systolic BP HbA1c	Positive (significant)	None	N/A
19	Jindal D	India	Descriptive	Not reported	Not reported	DM, HTN	None	N/A	None	N/A
20	Schwalm J-D	Colombia Malaysia	Cluster-randomized controlled trial	Not reported	1371	HTN	Framingham Risk Score Systolic BP LDL	Positive (significant)	None	N/A
21	Kengne AP (2009A)	Cameroon	Descriptive	6	925	DM, HTN, asthma, epilepsy	None	N/A	None	N/A
	Kengne AP (2009B)		Cohort				Fasting capillary glucose BP Body weight	Positive (significant)		
	Kengne AP (2008)	Cameroon	Cohort				Asthma attack frequency Hospital admission	Positive(significant/asthma attacks and hospital admission)	None	N/A
	Kengne AP (2009C)	Cameroon	Cohort				BP	Positive (significant)	None	N/A
22	Van de Vijver S (2013)	Kenya	Descriptive			CVD risk (obesity, HTN, DM)	None	N/A	None	N/A
	Van de Vijver S (2016)		Quasi-experimental		2764		BP	Positive (both groups)	None	N/A
23	Vedanthan R	Kenya	Descriptive	Not reported	Not reported	CVD, HTN, DM	None	N/A	Medicine availability Service availability	Positive
24	Katz I	South Africa	Cohort	20 clinics	618	HTN, DM, proteinuria	None	N/A	Detection Early referral Knowledge Follow up Staff workload Turnover	Mixed (It improved early detection and referral of high risk, poorly controlled patients. Its weaknesses include poor follow up due to poor existing health systems and inability to integrate into existing chronic disease services and overworked, poorly supported, poorly educated and frustrated primary health care team).
25	Chaowalaksakun P	Thailand	Qualitative	1	3116	DM	None	N/A	Model fit with situation and context	Positive
